# Low-Mass Liquid Crystalline Materials Blended in Recycled Thermoplastic Polyester Elastomer for Corrosion Inhibitor Application

**DOI:** 10.3390/polym13183188

**Published:** 2021-09-20

**Authors:** Chun-Jui Chen, Bo-Wei Huang, Po-Jung Tseng, Zhi-Yu Yang, Xiang Huang, Syang-Peng Rwei, Hsiu-Hui Chen

**Affiliations:** 1 Department of Molecular Science and Engineering, National Taipei University of Technology, No. 1, Sec. 3, Zhongxiao E. Rd., Taipei 106, Taiwan; fcps974210@yahoo.com.tw (C.-J.C.); h103330800@gmail.com (B.-W.H.); a123bd2015@gmail.com (P.-J.T.); andyang821222@gmail.com (Z.-Y.Y.); f10714@ntut.edu.tw (S.-P.R.); 2 Institute of Organic and Polymeric Materials, Research and Development Center of Smart Textile Technology, National Taipei University of Technology, Taipei 106, Taiwan; 3 College of Materials Science and Engineering, Shenzhen University, Shenzhen 518060, China; oyx@szu.edu.cn

**Keywords:** recycled polyethylene terephthalate, liquid crystal, corrosion inhibitor

## Abstract

In this work, the development and application of multicomponents obtained from recycled polyethylene terephthalate (*r*-PET) waste and monotropic liquid crystals as anticorrosion coatings are reported. The *r*-PET raw material was alcoholyzed and reproduced as a thermoplastic polyester elastomer (TPEE) with different amounts (n%, n = 0, 1, 3, and 5) of 1,6-hexanediamine (HDA). Then, a fluorine-containing liquid crystal (4-cyano-3-fluorophenyl 4-ethylbenzoate (4CFE)) was incorporated into the TPEE mixture via solvent blending to modify and enhance the water resistance. The adhesion behavior of the coating on glass and iron substrates was evaluated by cross-cut tests and immersion tests in aqueous NaCl. In the corrosion resistance measurements, all of the coating samples fabricated with 10 ± 1 mm thickness were less active toward electrochemical corrosion (*P*_EF_% > 99%) than the bare iron plate, indicating that our work provided better protection against corrosion of the iron plate.

## 1. Introduction

Metal corrosion is a common problem that is responsible for serious financial losses and even disasters. Anticorrosion measures, usually in the form of protective coatings on metal surfaces, need to be compatible with widely varying environmental factors. To inhibit or prevent corrosion, film coating is a useful approach to forming a stable protective layer on metallic surfaces, e.g., by using graphene [1,2,3], inert metal [4,5], or discotic columnar liquid crystals (DCLCs) [6,7]. A paint consisting of polymers that adheres to the surface well and prevents corrosion may be referred to as a polymeric corrosion-resistant coating. This type of coating includes urethane [8], polyvinyl chloride [9], and nylon [10]. If the coating substrate were obtained from a postconsumer commingled polymer (PCCP), it would represent a good solution to resolve the problem of waste pollution.

One of the main components of polymers available from waste pollution is poly(ethylene terephthalate) (PET), the most widely produced and used polymer worldwide [11,12]. Compared to burning, recycling/reusing PET can reduce the amount of waste and decrease environmental concerns. A new anticorrosion recycling route used beverage bottles made of PET to form a surface coating material for automobile fuel tanks [13]. However, the framework of the recycled polymer was changed after numerous processing cycles, resulting in poor mechanical properties compared to the original polymer. It seems that blending is the simplest and most convenient strategy to overcome these problems, because waste plastics are converted into composites during blending [14]. Various approaches, including physical/chemical recycled PET (*r*-PET), provide a useful and environmentally friendly method to produce a value-added product for reuse in anticorrosion coating materials. However, *r*-PET materials have no conductive properties, which makes them difficult to apply in anticorrosion coating. Nonetheless, Saidi et al. developed an anticorrosion coating using waste PET blended with different weight ratios of epoxy resin [15]. Atta et al. utilized polyurethane, which was obtained by glycolyzing *r*-PET into oligomers, as a protective coating in steel pipelines and observed the chemical resistance of the coating material in various aqueous solutions [16]. Nevertheless, *r*-PET blended with liquid crystals has seldom been reported as an anticorrosion material. Rod-like liquid crystals with nematic or smectic phases have been combined with polymers in applications such as electro-optic displays, switchable windows, and light shutters because of their optical anisotropy properties, through which the polymer provides confinement and mechanical reinforcement for functional liquid–crystalline components [17,18,19,20]. For example, LC droplets embedded in a polymer matrix can be tuned using an external field of polymer stabilized chiral liquid crystals (PSCLS) with nanoparticles that show the high dive frequency [21,22]; therefore, it can be used for a wide range of display and nondisplay applications.

In our work, in order to develop a wider application for *r*-PET, a series of thermoplastic polyester elastomer (TPEE) materials derived from *r*-PET were prepared and audaciously mixed with unstable liquid crystalline materials for property modulation. We expected that mixture would improve the drawbacks of both materials, including the lack of redox ability for anticorrosion application. The composite TPEE material, denoted as TPEEn%, consisted of a block copolymer named PET-block-PTMG, which was obtained in hard segments from *r*-PET and soft segments from poly(tetramethylene glycol) (PTMG), together with different amounts (n%, n = 0, 1, 3, and 5) of 1,6-hexanediamine (HDA), resulting in TPEE0%, TPEE1%, TPEE3%, and TPEE5%. The hard segment was prepared by alcoholysis of *r*-PET to yield lower-molecular-weight oligomers, and it was copolymerized with PTMG to increase the softness of the materials. Most importantly, HDA carrying two amino groups can give rise to hydrogen bonds between these segments to connect different frameworks [23]. On the other hand, 4-cyano-3-fluorophenyl 4-ethylbenzoate (4CFE), a fluorinated liquid crystal, is a known monotropic liquid crystalline molecule with a crystal-to-isotropic transition at 77 °C and an isotropic-to-crystal transition at 6 °C, which can be changed into the liquid crystal state from either an increase in the temperature of a solid or a decrease in the temperature of a liquid, but not both. According to previous studies, the fluorosubstituted liquid crystals were characterized by good optical and chemical stability, low viscosity, high specific resistance, and low threshold voltage due to their high polarity. The number of fluorosubstituents also affected the orientation of molecules [24,25]. Therefore, in 4CFE with fluorosubstitutents, the direction of the ester bonds was also affected by the strong dipole moment, resulting in strong positive dielectric anisotropy [26]. 4CFE was blended with *r*-PET to introduce anticorrosion properties to the system. This blend provides not only a new strategy to prevent the phenomenon of metal corrosion but a new concept for the reuse of *r*-PET. 

## 2. Materials and Methods

Materials: Recycled poly(ethylene terephthalate) (*r*-PET, Hong-Sheng Green Wealth Co., Ltd., ChangHua, Taiwan) and ethylene glycol (EG, commercial-grade) were purchased from Emperor Chemical Co., Ltd. (Taipei, Taiwan). Poly(tetramethylene glycol) (PTMG), M_n_ ~1000 g mol^−1^), 1,6-hexanediamine (HDA, 98% purity), titanium (IV) butoxide (TBT, 97%), and *d*-trifluoroacetic acid (*d*-TFA, 99.5%) were purchased from Aldrich (Westport Center Dr, St. Louis, MO, USA). 3-Fluoro-4-cyanophenyl-4-ethylbenzoate (>98%) was purchased from Tokyo Chemical Industry Co., Ltd. (Tokyo, Japan). Phenol (97%) was purchased from Aencore Chemical (Whitehorse Road, Surrey Hills, Australia). 1,1,2,2-Tetrechloroethane (97%) was purchased from Showa Chemical Industry Co. Ltd. (Shimo-Meguro Meguro-Ku, Tokyo, Japan). CHINOX *1010* (98%) was purchased from Double Bonding Partnership (Taipei, Taiwan).

**Scheme 1:**
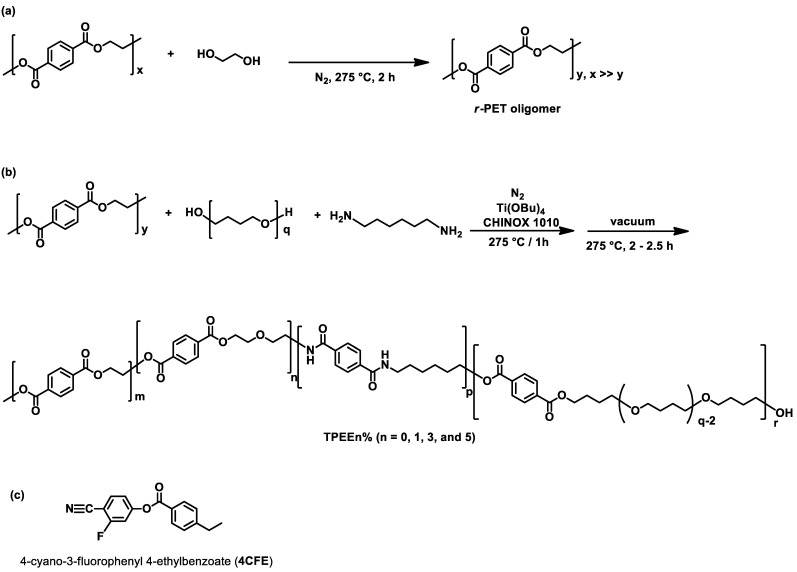
(**a**) Depolymerization of *r*-PET; (**b**) synthesis of TPEEn% copolymers; (**c**) the structure of 4-cyano-3-fluorophenyl 4-ethylbenzoate (4CFE).

**Figure 1:**
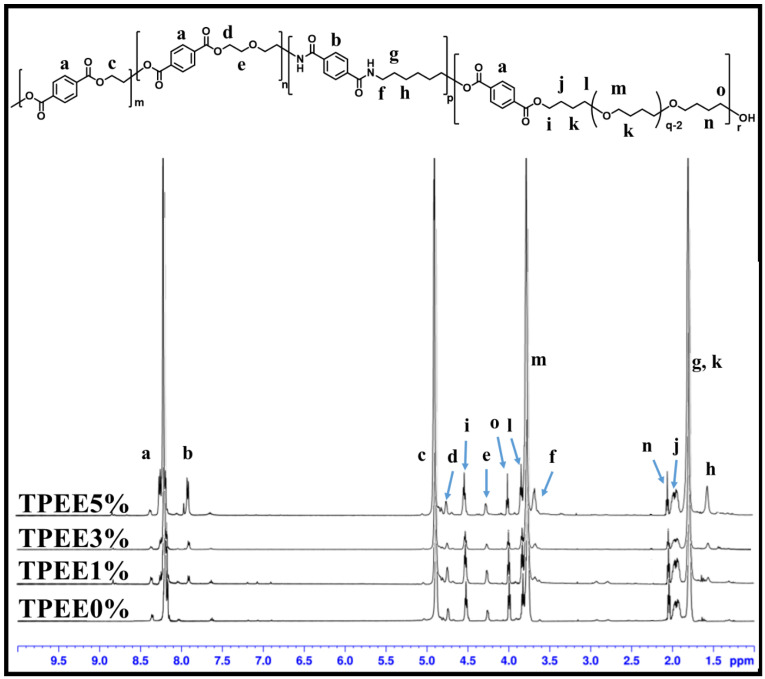
^1^H NMR spectra of TPEEn% (n was different amounts of HDA).

**Scheme 2:**
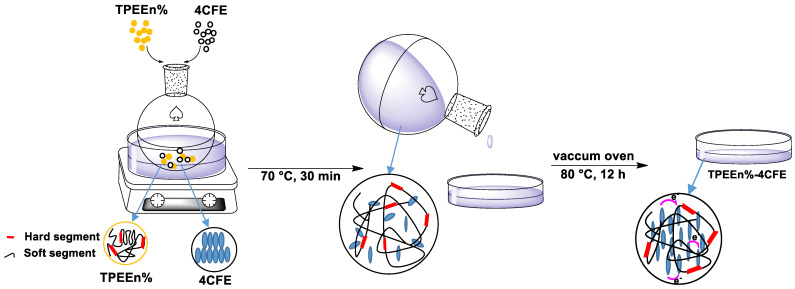
Preparation of TPEEn%-4CFE.

Methods: Preparation of TPEEn% and TPEEn%-4CFE Films: TPEEn% was synthesized via depolymerization of *r*-PET (Scheme 1a), transesterification, amidation for 1 h, and then polycondensation for 2–2.5 h (Scheme 1b). The *r*-PET chip (420 g) and EG (20 g, 0.32 mol) were charged into a 2 L stainless steel reactor, and the mixture was heated to 275 °C under nitrogen for 2 h for the depolymerization reaction. Then, PTMG (180 g, 0.18 wt%) and different amounts of HDA (1%, 3%, and 5%) were added to the reactor, with 400 ppm TBT as the catalyst and 1000 ppm CHINOX *1010* as the antioxidant for transesterification and amidation. After stirring for 1 h, the pressure was reduced to 30 mbar, maintained for 30 min, and then reduced to 1 mbar for 1.5–2 h to remove the byproducts of polycondensation. The initial stirring torque was recorded to be approximately 85 torque, and the reaction was finished when it reached approximately 1.2 times the initial stirring torque (Scheme 1b). TPEE0%, TPEE1%, TPEE3%, and TPEE5% were obtained, and the chemical structures were identified using ^1^H NMR, as shown in Figure 1 [23]. The process for preparing the blends of TPEEn%-4CFE was: a 1:1 (*w*/*w*) ratio of TPEEn% and 4-cyano-3-fluorophenyl 4-ethylbenzoate (4CFE) (Scheme 1c and Scheme 2) was mixed and dissolved in 10% phenol/1,1,2,2-tetrachloroethane (3:2, *w*/*w*) solution at 70 °C for 30 min. 

Characterization: Fourier transform infrared (FT-IR) spectra were measured with a PerkinElmer Spectrum One (Cinnaminson, NJ, USA) in attenuated total reflection (ATR). The sample was analyzed at a resolution of 4 cm^−1^ with 16 scans over the wavenumber range of 650–400 cm^−1^. Differential scanning calorimetry (DSC) was performed using a PerkinElmer DSC 800 (Waltham, MA, USA) Calorimeter; then, the prepared sample (~5 mg) was heated from room temperature to 110 °C and then cooled at the same rate of 10 °C min^−1^ under a nitrogen atmosphere using aluminum pans. Adhesion testing of the coating material on iron substrate were evaluated by cross-cut test. The coating film was cut into 100 square sections by penknife. Then, the sample was gently brushed clean along the diagonal directions 5 times each using a brush pen over the cuts, and Permacel tape was applied to the film and peeled to remove the cut sections. The adhesivity was measured by counting the number of remaining sections using the following method:

(1) $$\mathrm{Adhesivity}=\frac{\mathrm{Detached}\;\mathrm{square}\;\mathrm{section}}{\mathrm{Initilal}\;\mathrm{square}\;\mathrm{section}}\times \;100\%$$

The corrosion resistance was investigated using a VoltaLab and the method of *dc* polarization, a commonly used technique for corrosion studies. A more positive potential (*E_corr_*), a lower corrosion current (*I_corr_*), and a higher polarization resistance (*R_p_*) indicate better corrosion resistance. Polarization tests were conducted after immersion in a corrosive medium (3.5 wt% aqueous NaCl solution).

The contact angle was measured with a contact angle tester Phoenix 300 (S.E.O, Yongin-si, Korea) using water as the testing liquid. Sets of sessile droplets of testing liquid were placed on the surface and measured within 15 s. The water vapor permeability rate was measured using the Permatran-W 3/61 according to the ASTM F-1249. The sample was sealed into incubator with 25 °C at 100% relative humidity. Then, the results were recorded and analyzed automatically by the water vapor transmission rate tester.

## 3. Results and Discussion

**Figure 2:**
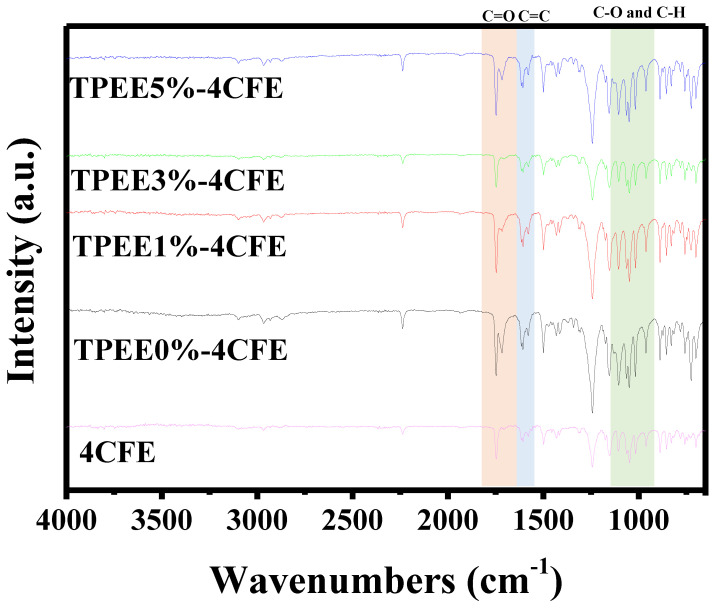
FT-IR of TPEEn%-4CFE and 4CFE.

FT-IR analysis: The FTIR spectral data of TPEEn%-4CFE and 4CFE are shown in Figure 2 and Table 1. The main signals of 4CFE were at 1746 cm^−1^ (C=O, amide group), 1579 and 1606 cm^−1^ (C=C, aromatic ring), and 1244 cm^−1^ (C-O, ester group). While the mentioned bands for the N-H absorption of the amide group in TPEEn% blended with LC were changed, the characteristic signals of TPEEn% were observed, with the aromatic ring (C=C) at approximately 1578–1607 cm^−1^ and the C=O of the amide group at ~1747 cm^−1^. There were also bands at approximately 1095 cm^−1^ (C-H asymmetric stretching) and 1017 cm^−1^ (C-O of the amide group). A band corresponding to N-H absorption was found at 1716–1717 cm^−1^, and the presence of N-H bands plus an unusually low value for C=O suggests the presence of an amide functional group. Noticeably, TPEE3%-4CFE did not show an N-H stretch, which suggests the formation of hydrogen bonding (H-bonding) between TPEEn% and 4CFE, making the secondary amide behave as a tertiary amide in TPEE3%-4CFE. TPEE3%-4CFE is proposed to have optimal blending between these two materials.

**Figure 3:**
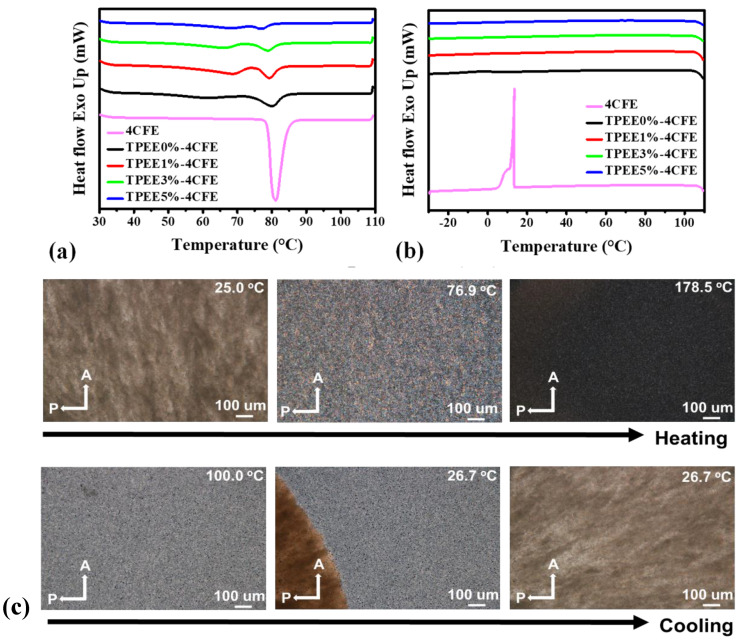
Thermal characterization of 4CFE and TPEEn%-4CFE and the POM texture of TPEE3%-4CFE. (**a**) DSC first-heating thermograms, (**b**) DSC first-cooling thermograms, and (**c**) the mesophase of TPEE3%-4CFE from 100 to 25 °C with analysis for POM (bright parts are 4CFE and black parts are TPEEn%).

Mesomorphic behavior: Differential scanning calorimetry (DSC) and polarized optical microscopy (POM) were used to characterize the phase behavior of the liquid crystals, as shown in Figure 3. The melting point data suggest that as the amount of HDA increased, the melting points were shifted toward lower temperatures. The neat LC gave a large endotherm with a peak temperature at ~81 °C, corresponding to the melting of the crystals. The peak shifted to a lower temperature (<80 °C) during the heating process as more HDA was added, as shown in Figure 3a. The low-molecular-weight LC molecules entered the crystalline domains of *r*-PET, softening it and increasing its flexibility. In the cooling process, no exothermic peak was found for all composites, which suggests that the crystallinity decreased. The crystalline degrees of TPEEn%-4CFE were 28–55%, as shown in Table 1 and Figure 3b. The film was heated to over the isotropic temperature at 100 °C as shown in Figure 3c, which produced a colorless surface and then presented a dark image under the measurement of POM with the analyzer plate. When the temperature was cooled to room temperature, 25 °C, a cloudy surface and a crystalline image of 4CFE appeared. The TPEEn% films to which LC materials were not added only showed cloudy surfaces at different temperatures.

**Figure 4:**
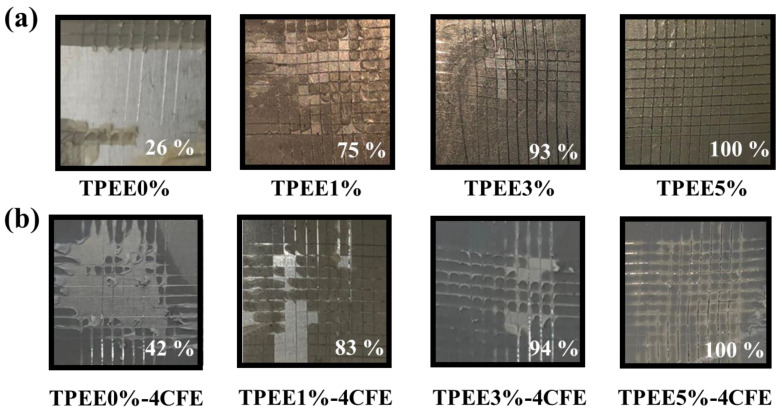
Adhesion testing of (**a**) TPEEn% and (**b**) TPEEn%-4CFE coated on an iron surface and treated by the cross-cut method.

Adhesion analysis: The cross-cut test is a simple and easily applied method for evaluating the adhesion of single- or multicoat systems. We created a lattice pattern in the film by cutting the substrate into 100 square sections with a penknife. Then, the sample was gently brushed clean along the diagonal directions 5 times each using a brush pen over the cuts, and Permacel tape was applied to the film and peeled to remove the cut sections. Finally, the unremoved sections were counted to assess the adhesion ability. Figure 4 shows the test results before blending 4CFE into the matrix, where TPEE0% and TPEE1% retained ~26% and ~75% of the materials on the surface, respectively. After 4CFE was added, the adhesion properties of TPEE0% and TPEE1% significantly increased by ~42% and ~83%, respectively. The results showed a substantial improvement because of the increase in mechanical strength after blending. TPEE3%-4CFE and TPEE5%-4CFE showed excellent adhesion ability before and after blending the LC moiety; ~>93% remained.

**Figure 5:**
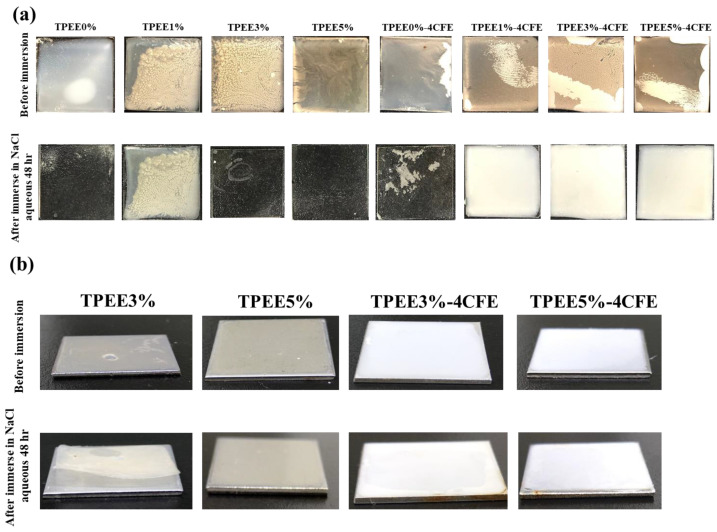
Adhesion testing of TPEEn% and TPEEn%-4CFE coated on (**a**) glass and (**b**) iron substrates and immersed in aqueous NaCl for 48 h.

To clarify the effect of the aging phenomena on the adhesion performance, an adhesion test immersed in aqueous NaCl for 48 h on glass and iron substrates was performed, as shown in Figure 5. As seen in Figure 5a, only the adhesion of the TPEEn% coating on the glass surface was poor. After blending with 4CFE, the adhesive ability of TPEE0%-4CFE on the glass was improved except for TPEE0%-4CFE, indicating that the addition of 4CFE can promote the interfacial adhesion ability of TPEEn%. In Figure 5b, an iron substrate was used. The results showed that the coating on the iron was completely peeled off after immersion in NaCl aqueous for 48 h in low-content HDA of TPEEn% and TPEEn%-4CFE (n = 0, 1); on the contrary, the adhesion property on the substrate was increased for the high-content HDA (n = 3, 5).

**Figure 6:**
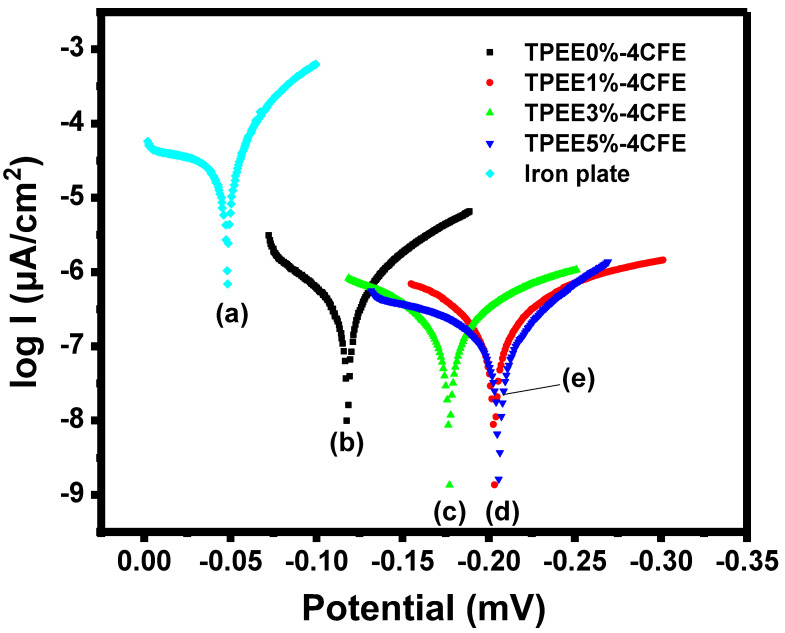
Tafel plot of the corrosion protection of (**a**) iron plate, (**b**) TPEE0%-4CFE, (**c**) TPEE3%-4CFE, (**d**) TPEE1%-4CFE, and (**e**) TPEE5%-4CFE.

Potentiodynamic measurements: The test results were obtained by extrapolating Tafel plots from both the cathodic and anodic polarization curves for the respective corrosion processes [27,28]. Extrapolating the cathodic and anodic polarization curves to their point of intersection provided both the corrosion potential and the corrosion current. Corrosion resistance studies were performed on samples with 10 ± 1 mm-thick coatings that were immersed in a corrosive medium for 30 min. Tafel plots for the iron plate, TPEE0%-4CFE, TPEE1%-4CFE, TPEE3%-4CFE, and TPEE5%-4CFE are shown in Figure 6, and the corresponding data are listed in Table 2. Figure 6a shows Tafel plots for the bare iron plate, and Figure 6b–e shows the TPEEn% with LC blending after annealing treatment. The coated iron electrodes gave corrosion potentials of *E_corr_* = −0.71 V, −0.66 V, −0.68 V, and 0.65 V for TPEE0%-4CFE, TPEE1%-4CFE, TPEE3%-4CFE, and TPEE5%-4CFE, respectively, which were more positive than that of the bare iron electrode, for which *E_corr_* = −0.90 V. Moreover, the corrosion currents (*I_corr_*) of the TPEEn%-4CFE iron electrodes were ca. 2.87–7.09 × 10^−8^ A cm^−2^, which were significantly lower than that of the bare iron plate (8.78 × 10^−5^ A cm^−2^). The protection efficiency (*P*_EF_%) values were estimated using the following Equation [29,30]:

$$P_{\mathrm{EF}}\%=100\;\pm \;\frac{I_{\mathrm{corr}}\;-\;I_{\mathrm{corr}\;(C)}}{I_{\mathrm{corr}}}$$

where *I_corr_* and *I_corr_*_(C)_ are the corrosion current values in the absence and presence of the coating, which were determined from the Tafel plots.

All TPEEn%- 4CFE coatings showed *P*_EF_% > 99%, suggesting that they were less active toward electrochemical corrosion than bare iron plates. The above electrochemical measurement results show that the TPEEn%-4CFE coating provided protection against corrosion of the iron plate.

**Figure 7:**
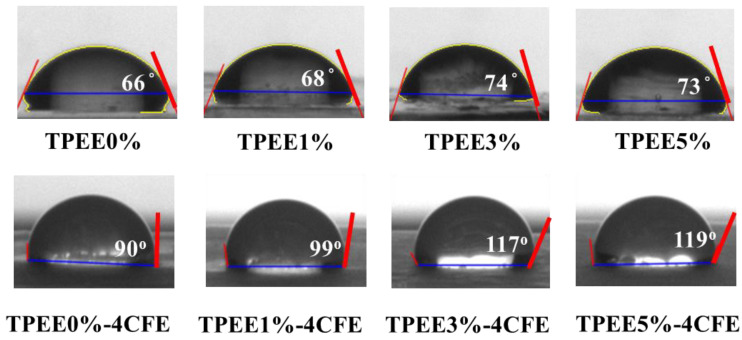
The contact angle results for the TPEEn% and TPEEn%-4CFE membranes.

Mechanism of anticorrosion for TPEEn%-4CFE:The corrosion enhancement effect of TPEEn%-4CFE is attributed to its nonpolar composition (self-assembling behavior of liquid crystal), giving a hydrophobic surface (Figure 7). After blending, the contact angle was increased from 66°–74° (TPEEn%) to 90°–123° (TPEEn%-4CFE), suggesting that the introduction of 4CFE within the polymeric matrix increased the roughness of the surface and then enhanced its waterproof properties [31,32].

**Figure 8:**
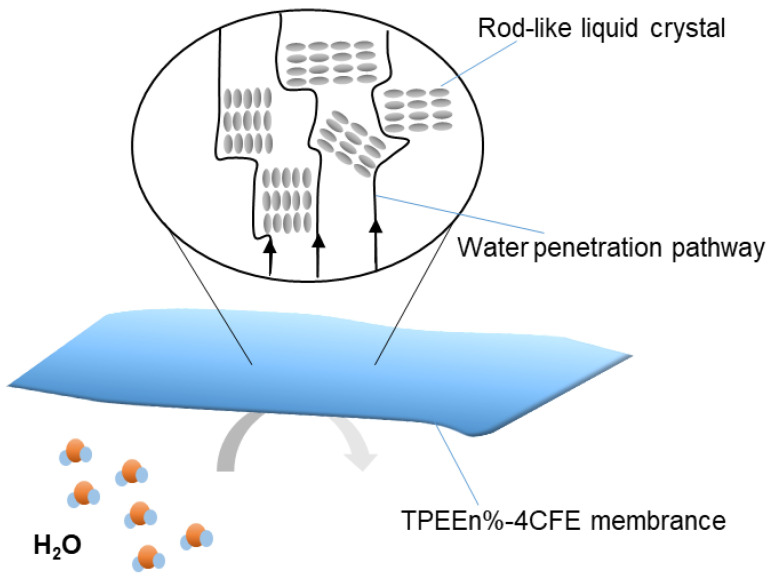
Schematic representation of water following a tortuous path through the TPEEn%-4CFE materials.

Apart from the hydrophobic surface, the water vapor transmission rate (WVTR) of the coating material also affects the anticorrosion behavior. Compared to pristine TPEEn%, the TPEEn%-4CFE membranes showed a decrease in H_2_O permeability, as shown in Table 3. The decrease in water permeability is attributable to the liquid crystal, which was confined in the TPEE network, acting as a barrier and providing tortuosity of pathways to prevent the diffusion of aggressive species from reaching the metal surface (Figure 8). The results illustrate that the incorporation of LC suppressed the diffusion of water molecules as a result of the increased diffusion length; the hydrophobicity of the TPEEn%-4CFE membranes further prohibited the adsorption of H_2_O molecules on the surface, and hence, enhancement of the anticorrosion properties was achieved.

## 4. Conclusions

In conclusion, multicomponents obtained from *r*-PET and rod-like liquid crystals by solvent blending were successfully developed into anticorrosion coating materials because of their hydrophobic surfaces and excellent attachment properties on metallic surfaces. From FT-IR analysis, it can be concluded that the sample was well blended via a convenient processing method. The adhesion properties on iron and glass substrates were determined by a cross-cut test and immersion in aqueous NaCl. Compared with regular *r*-PET, the TPEEn%-4CFE possessed more hydrophobic properties on the surface and lower water vapor permeability rate due to the influence of LC. After blending, the contact angle was increased from 66°~74° (TPEEn%) to 90°~123° (TPEEn%-4CFE), suggesting that the LCs within the polymeric matrix increased the roughness of the surface and then enhanced the waterproof properties. Furthermore, the liquid crystal, which was confined in the TPEE framework, acted as a barrier to decrease the diffusion of water. Hence, the anticorrosion properties of the TPEEn%-4CFE membranes were mainly due to the synergistic effect of hydrophobicity and the increased diffusion path, indicating that these films can be a great passivation layer for the application of corrosion-resistant coatings, which opens up a new application for *r*-PET in the near future. Although the liquid crystalline property was not directly observed in these mixtures, a great modulated property of *r*-PET was shown in our work. In future work, attempts can be made to blend different liquid crystalline materials with *r*-PET.

## Figures and Tables

**Table 1:** DSC data of 4CFE and TPEEn%-4CFE.

Sample	1st Heating				1st Cooling		Xc ^a^ (%)
	T_m_ (°C)	ΔH_m1_ (J/g)	T_m2_ (°C)	ΔH_m2_ (J/g)	T_cc_ (°C)	ΔH_cc_ (J/g)	
4CFE	81.1	89.1			13.6	62.5	-
TPEE0%-4CFE	61.9	7.1	80.1	14.4	−2.1	2.1	48.8
TPEE1%-4CFE	68.6	14.7	79.2	9.4	-	-	54.6
TPEE3%-4CFE	66.0	11.2	78.8	10.0	-	-	48.2
TPEE5%-4CFE	68.3	8.1	76.5	4.2	-	-	28.0

^a^ Xc = ∆H_m_/(W_f_ × ∆H_100_) × 100%

**Table 2:** Electrochemical corrosion measurements of a bare iron plate and TPEEn%-4CFE.

	Electrochemical Corrosion Measurements ^a^				
Materials	*E_corr_* (V vs. SCE)	*R_p_* (Ω cm^2^)	*I_corr_* (μAcm^−2^)	*P*_EF_ (%)	Thickness (mm)
Iron plate	−0.90	615.94	8.78 ×10^−5^	—	—
TPEE0%-4CFE	−0.71	3616.10	0.28 ×10^−8^	>99%	1.13 ± 0.002
TPEE1%-4CFE	−0.66	1327.40	0.71 ×10^−8^	>99%	1.13 ± 0.009
TPEE3%-4CFE	−0.68	1475.00	0.41 ×10^−8^	>99%	1.13 ± 0.004
TPEE5%-4CFE	−0.65	1344.40	2.54 ×10^−8^	>99%	1.13 ± 0.009

^a^ Saturated calomel electrode (SCE) was employed as a reference electrode.

**Table 3:** Vapor permeability of TPEEn% and TPEEn%-4CFE.

	TPEE0%	TPEE1%	TPEE3%	TPEE5%
Vapor permeability (gm/m^2^ day)	359.7	320.5	227.1	256.6
	TPEE0%-4CFE	TPEE1%-4CFE	TPEE3%-4CFE	TPEE5%-4CFE
	39.3	307.9	155.9	215.8

## Data Availability

The data presented in this study are available on request from the corresponding author.
